# 683. People Can Change! The Impact of Pharmacist-Led Interventions on Reducing Antibiotic Self-medication in Rural Tamil Nadu, India

**DOI:** 10.1093/ofid/ofaf695.222

**Published:** 2026-01-11

**Authors:** Suhail Hassan Jalal, Roshni Murali, Giris Sharma, Libis Linga Sivaraj

**Affiliations:** The Tamil Nadu Dr. M.G.R. Medical University, Chennai, Tamil Nadu, India; The Tamil Nadu Dr. M.G.R. Medical University, Chennai, Tamil Nadu, India; The Tamil Nadu Dr. M.G.R. Medical University, Chennai, Tamil Nadu, India; The Tamil Nadu Dr. M.G.R. Medical University, Chennai, Tamil Nadu, India

## Abstract

**Background:**

Inappropriate antibiotic use, driven by self-medication and community-level misconceptions remains as a major contribution to antimicrobial resistance in India. This study assesses the impact of pharmacist-led, community-based education in the reduction of self-medication practices and antibiotic expectations in rural South India.

**Figure:**
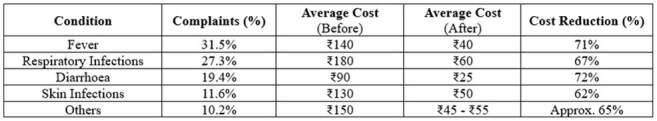
Cost-Saving Outcomes

**Figure:**
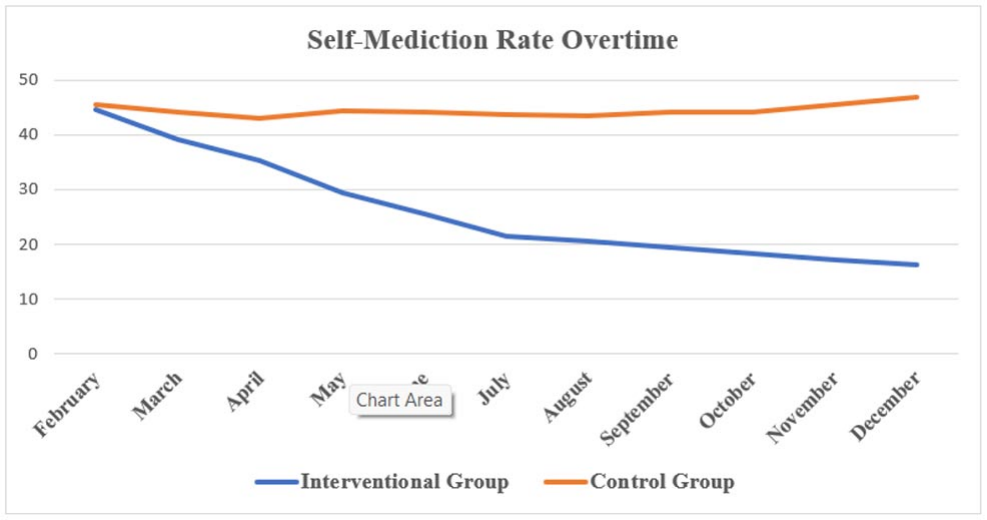
Self Medication Rates

**Methods:**

We conducted a controlled pre-post study between July to December 2024 in two demographically similar rural villages of Tamil Nadu, India. The intervention village was provided with a multi-modal educational program, involving weekly awareness sessions, illustrated handouts and social media campaigns. The social media campaign aimed local community groups to promote the importance of proper health-seeking behaviours and to reduce self-medication. The control village received standard care. The primary outcomes included changes in self-medication behaviour and antibiotic expectations, measured using pre- and post-intervention knowledge, attitudes and practices (KAP) surveys as well as self-medication rates from local pharmacy data.

**Figure:**
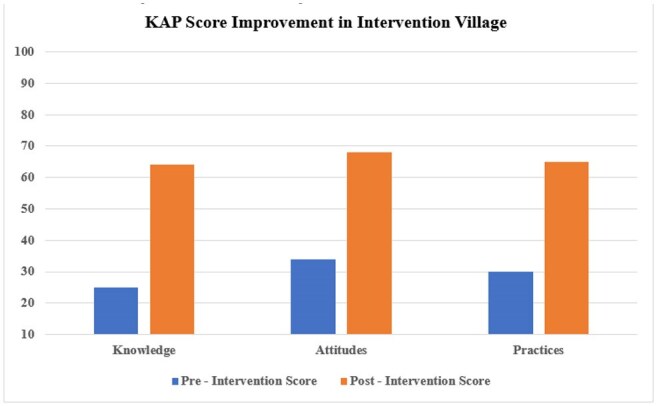
KAP Score Improvement Comparison

**Results:**

A total of 827 participants (412 in the intervention and 415 in the control village) completed both the pre and post-surveys. In the intervention village, self-medication practice was significantly reduced by 63.6% (from 44.5% to 16.2%, p < 0.001), while the control village showed no significant change. Antibiotic expectation during fever episodes dropped from 62% to 24% in the intervention village (p < 0.001). Mean knowledge scores increased by 41% in the intervention group (p < 0.001). Post-campaign, 88% of participants in the intervention village recognized pharmacists as a trusted health resource. Analysis of pharmacy records showed a 67–72% reduction in average treatment costs for common self-treated conditions.

**Conclusion:**

The study highlights the significant contributions pharmacists can make in promoting antimicrobial stewardship and reducing AMR at the grassroots level. This intervention model, which uses digital and social media platforms, could be applied in other rural areas & communities which could make people understand the importance of AMR.

**Disclosures:**

All Authors: No reported disclosures

